# Supplementary material for: Complex crater formation by low energy impactors
Source: PLoS One. 2025 Nov 6;20(11):e0326628. doi: 10.1371/journal.pone.0326628 (PMC12591455; doi:10.1371/journal.pone.0326628)
Supplement: S1 File — Modelling of granular projectile impact. (PDF) [file pone.0326628.s001.pdf]

# S1 File - Supporting Information for article "Complex crater formation by low energy impactors"

## Modelling of granular projectile impact

The study of granular impacts, particularly the formation of craters resulting from the collision of granular projectiles with granular beds, has attracted considerable attention due to its relevance in both natural and industrial contexts. Accurately capturing the complex dynamics of such systems typically requires sophisticated numerical tools capable of modeling large numbers of interacting discrete elements.

However, the existing simulation platforms presented significant limitations for our specific objectives. On one hand, high-fidelity discrete element method (DEM) frameworks are often computationally intensive, complex to configure, and inflexible with regard to integrating custom analysis pipelines. On the other hand, simpler or more abstracted models, while computationally efficient, often lack the physical resolution necessary to reproduce the detailed morphological features observed in controlled laboratory experiments—such as crater depth, radius, and central peak formation.

Faced with this gap between overgeneralized simplicity and overly complex simulation environments, we developed a tailored computational methodology designed to balance physical realism with computational efficiency. This custom framework allowed us to systematically simulate granular impacts in two dimensions and to extract quantitative morphological characteristics from each impact event. Moreover, it enabled the incorporation of an evolutionary optimization algorithm to calibrate model parameters against experimental data, providing a practical and adaptable approach to studying granular cratering phenomena.

The key software used in this study was designed to individually track each sand grain (Howling Moon Software 2024), recording its properties, such as position and rotation, during interactions with neighboring grains in contact. This approach allows for a detailed analysis of granular behavior at the microscopic scale, ensuring precision in the simulation of forces and displacements. Furthermore, the software was developed to guarantee the conservation of fundamental properties, including mass, linear momentum, angular momentum, and energy, thereby ensuring the physical consistency of the results. Standard tests, as the dilatancy model test (Liang et al. 2020), were also performed for adjustment and calibration, ensuring that the outcomes accurately reflected the modeled physical conditions.

## S1. Modeling and Physical Parameters

Particles were represented as rigid two-dimensional bodies with circular, pentagonal, or hexagonal geometries. Each particle was assigned a radius that varied randomly within a predefined interval, representing the intrinsic heterogeneity of real granular systems. The mechanical properties of the particles—namely, the coefficient of restitution (elasticity) and the coefficient of static friction—were kept constant for all particles within a given simulation.

The system was modeled in 2D for computational simplicity, while retaining the essential mechanical interactions involved in the impact process and the redistribution of particles.

## S2. Simulation Procedure

Each individual simulation, hereafter referred to as a "drop," consisted of releasing a granular projectile onto a bed of initially static particles (Figure S1). The projectile was composed of a cluster of particles rigidly grouped together, simulating a coherent impact on the granular surface.

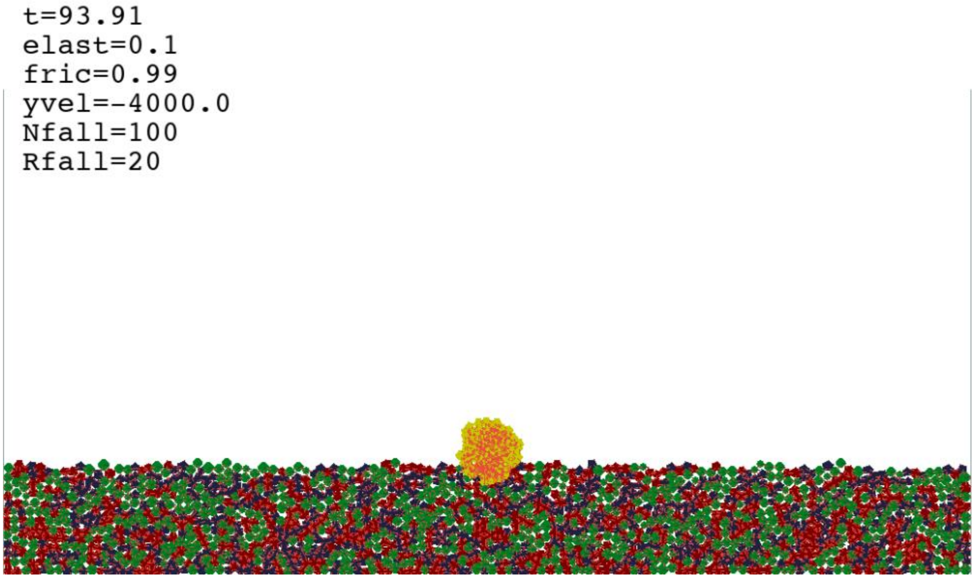


**Fig. S1. Example frame of the simulation.** A frame of the simulation at the moment the falling material contacts the granular bed. Simulation parameters and elapsed time are also displayed.

The simulation evolved over time until all particles reached a state of rest, defined as the condition in which the total kinetic energy of the system fell below a predefined threshold. This served as the stopping criterion, at which point the system was considered to be in a steady state.

Simulations were conducted using non-dimensional units, normalized by the mean grain diameter and gravitational acceleration. Several correlations between input parameters and resulting crater morphology were identified, with the most significant discussed in the following sections.

## S3. Surface Extraction and Morphological Feature Analysis

At the end of each simulated impact (“drop”), the surface profile of the granular bed was analyzed to extract key morphological features of the resulting crater. The process began with identifying the subset of particles located at the exposed upper surface of the granular medium. This was done using a neighbor-counting criterion: particles with a low number of neighboring contacts were classified as surface particles. Once identified, the spatial coordinates of these particles were used to reconstruct a smooth and continuous surface through bicubic spline interpolation. This interpolation step mitigates discretization artifacts intrinsic to the granular nature of the medium, allowing for the extraction of continuous morphological descriptors.

From this interpolated surface, five primary morphological descriptors were extracted:

- **Crater radius** ($r_{crater}$): Horizontal distance from the center of impact to the outer rim of the disturbed region.
- **Depression radius** ($r_{depression}$): Extent of the central depressed area.
- **Central peak height** ($h_{peak}$): Distance from the lowest point of the depression to the apex of the central peak.
- **Crater wall height** ($h_{crater}$): Vertical distance from the depression bottom to the crater rim crest.
- **Depression depth** ($l_{depression}$): Vertical distance from the undisturbed surface to the bottom of the depression.

These features were selected for their relevance in characterizing crater geometry and their consistency with laboratory observations.

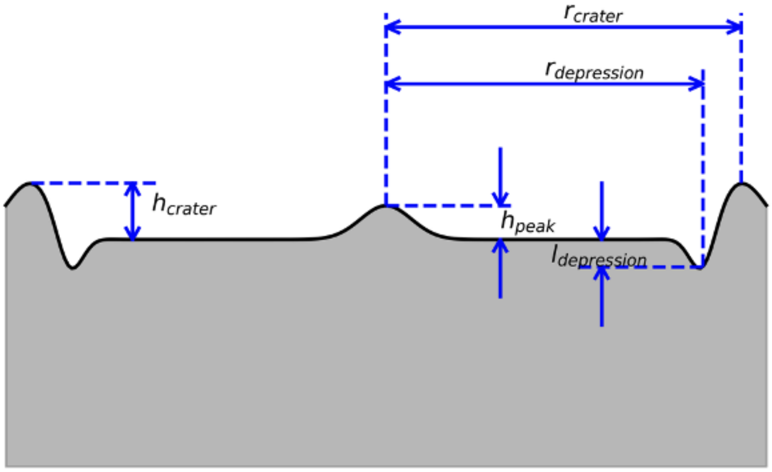


**Fig. S2. Crater morphology and its relevant features.** Schematic representation of the crater profile and key morphological features extracted from the interpolated surface. The crater radius ($r_{crater}$) is the horizontal distance from the center of impact to the outer rim of the disturbed region. The depression radius ($r_{depression}$) defines the extent of the central depressed area. The central peak height ($h_{peak}$) is measured from the lowest point of the depression to the apex of the central peak. The crater wall height ($h_{crater}$) corresponds to the vertical distance from the depression bottom to the outer rim crest. The depression depth ($l_{depression}$) is the vertical distance from the undisturbed surface to the bottom of the central depression.

## S4. Batch Execution and Statistical Aggregation

To mitigate stochastic effects arising from the random distribution of particle radii and spatial discretization, each parameter set was subjected to a series of repeated simulations, collectively referred to as a *batch*. The morphological features were computed for each individual drop and statistically aggregated using the mean, representing the average behavior of the system under that specific set of parameters.

Figure S3 presents the distribution of central peak height and depression depth. As shown, no correlation was observed between these two quantities, suggesting that the simulation process does not induce artificial dependencies between independent features.

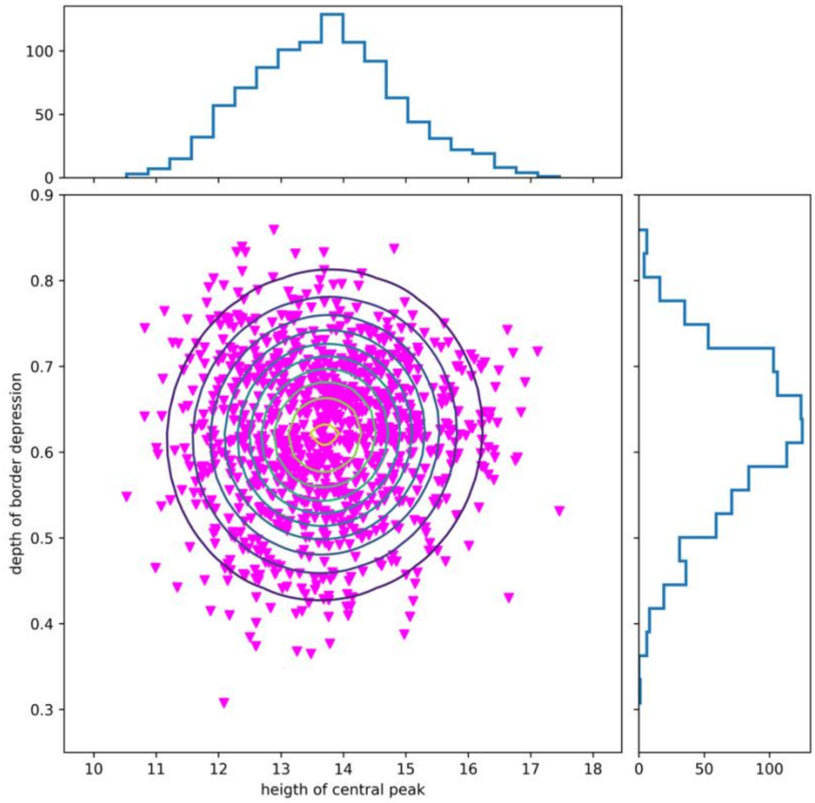


**Fig. S3. Distribution of obtained central height and depression depth.** Distribution of central peak height and depression depth from a batch of 1000 simulations, with corresponding histograms. The central panel shows contour levels of the best-fit bivariate normal distribution.

A correlation was observed between the friction coefficient among grains and the height of the central peak. This behavior may result from the difficulty of grains in the peak region to slide, which would reduce the height of the resulting peak. Another correlation was observed between the terminal vertical velocity of the projectile and the central peak height. It is hypothesized that this effect results from the interaction between the incoming grains and the static surface grains. Unrealistically high impact velocities can cause excessive penetration and peak deformation, which is not typically observed in meteorite impacts, where explosive effects dominate.

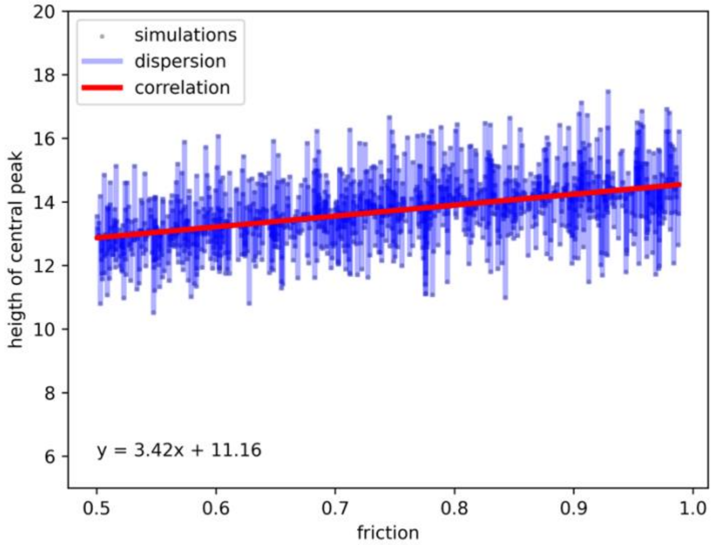


**Fig. S4. Correlation between friction and central peak height.** Correlation between friction coefficient and central peak height. Blue regions represent the dispersion of simulation results. A correlation function is shown in the bottom-left corner (expressed in grain units; see text for details).

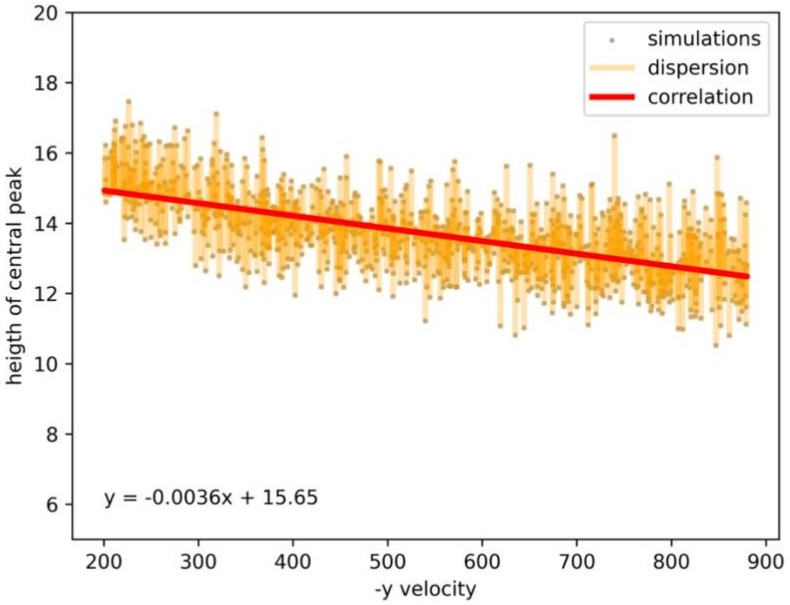


**Fig. S5. Correlation between impact velocity and central peak height.** Correlation between impact velocity and central peak height.

## S5. Optimization via Genetic Algorithm

The final step consisted of calibrating the simulation parameters to reproduce the experimentally observed results. A classical genetic algorithm was employed, operating on the following input parameters:

- Range of particle radius variation;
- Coefficient of friction;
- Coefficient of restitution.

The objective function of the algorithm was defined as the minimization of the mean squared error between the simulated morphological features (after batch averaging) and the values measured in laboratory experiments. The algorithm executed successive generations of batches, applying selection, crossover, and mutation operators, with elitism to preserve the best-performing solutions.

A variation of the classical genetic algorithm was developed, using pure elitism for selection, uniform crossover and mutation, and real-valued gene encoding. The algorithm maintained 5% elitism, discarded 5% of the worst individuals, and introduced 1% mutants in each generation. A population size of 100 individuals was used. The results demonstrated stable convergence and reproducibility of the optimized parameters.

Once the desired parameter values were successfully adjusted, the model demonstrated a robust capacity to reproduce the expected behavior within the defined constraints. This outcome validates the reliability of the implemented optimization process and highlights the model's potential for accurate predictions in scenarios of interest. The achieved parameter set represents an optimal solution, ensuring consistency with theoretical expectations and aligning with the objectives initially established for this study.

Figure S6 illustrates the evolution of the fitness function during the first 200 generations of the genetic algorithm. The fitness function represents the degree of agreement between the simulated crater morphology and the experimental reference values. A consistent downward trend indicates that the algorithm successfully identified progressively better parameter combinations with each generation. The curve stabilizes after a certain number of iterations, suggesting convergence toward an optimal or near-optimal solution. This behavior demonstrates the effectiveness of the evolutionary approach in navigating the parameter space and minimizing the objective error function.

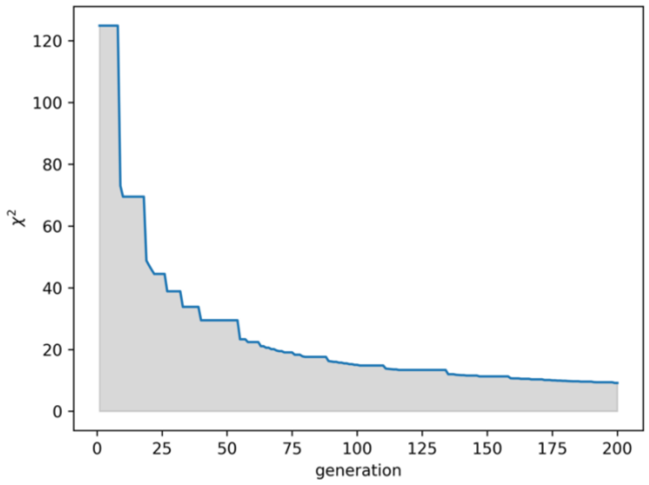


**Fig. S6. Convergence of genetic algorithm.** Evolution of the fitness function over the first 200 generations of the genetic algorithm.

Figures S7 and S8 present the results obtained after the optimization process. Figure S7 displays the normalized errors for each morphological parameter, revealing that all deviations between fitted and target values fall within acceptable limits. This confirms the robustness of the fitted solution and the ability of the model to reproduce the desired crater characteristics. Figure S8 complements this analysis by showing the fitted crater profile. On the left, the black line represents the 2D profile derived from the optimized parameters, while the envelope represents the maximum deviation observed. On the right, a cross-section of the corresponding 3D crater model is shown, with the red line highlighting the 2D profile. Together, these visualizations validate the consistency between the simulated and reference geometries, further reinforcing the reliability of the optimization.

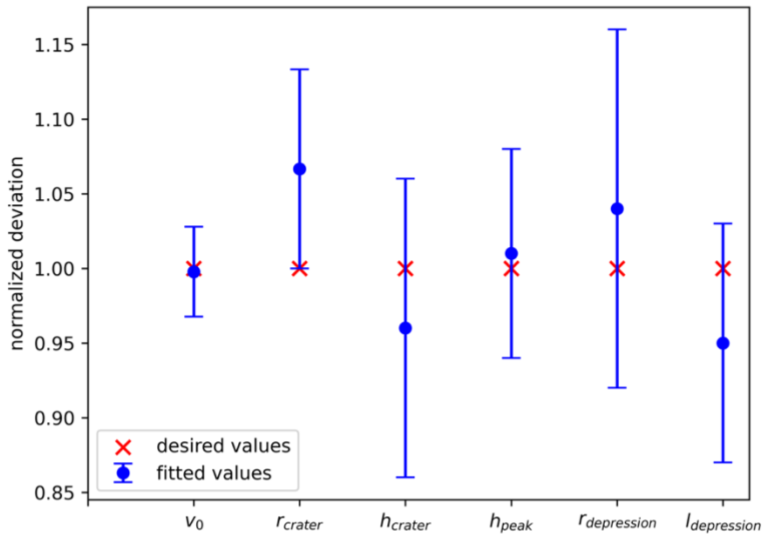


**Fig. S7. Optimized parameters.** Normalized errors for each target parameter after optimization.

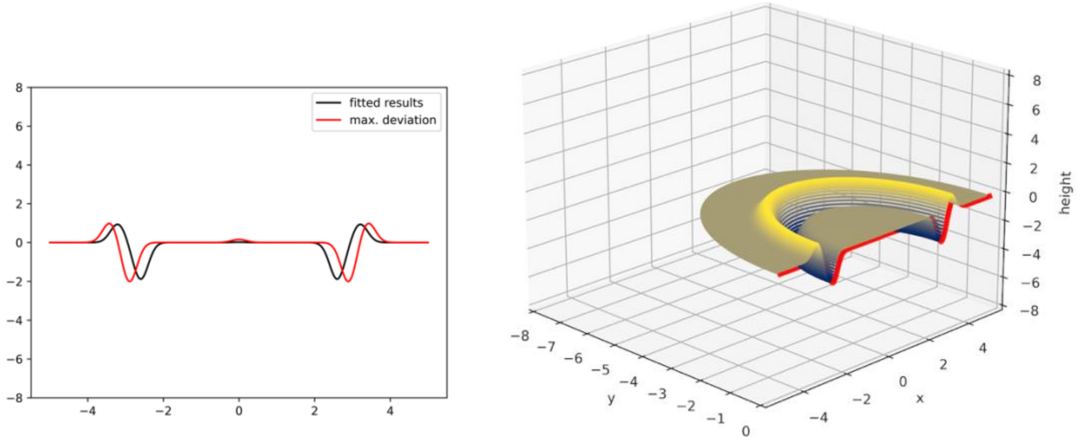


**Fig. S8. Simulated crater profile.** (Left) Crater profile for the fitted results (black line), along with the profile for the maximum deviation. (Right) Cross-section of the 3D crater model generated from the fitted parameters, with the red line indicating the 2D profile.

Tables S1 and S2 summarize the numerical results of the optimization process. Table S1 compares the target (experimental) values of the five morphological features with the values obtained through simulation using the optimized parameters. The fitted values align closely with their targets, typically within one standard deviation, indicating a high degree of accuracy. Table S2 presents the final values of the input parameters identified by the genetic algorithm, including impact velocity, friction coefficient, and restitution coefficient. These values define the configuration that best reproduces the experimental crater

morphology, and they serve as a validated reference for future simulations under similar conditions.

**Table S1. Morphological parameter values.** Target values of morphological descriptors compared to the corresponding optimized values obtained from the genetic algorithm.

| parameter | desired value | fitted value |
|---|---|---|
| $r_{\text{crater}}$ [cm] | 3.0 | 3.2±0.2 |
| $h_{\text{crater}}$ [cm] | 0.5 | 0.48±0.05 |
| $h_{\text{peak}}$ [cm] | 0.0 | 0.01±0.07 |
| $r_{\text{depression}}$ [cm] | 2.5 | 2.6±0.3 |
| $l_{\text{depression}}$ [cm] | 1.0 | 0.95±0.08 |

**Table S2. Input parameter values.** Final optimized values of the input parameters: impact velocity, coefficient of friction, and coefficient of restitution, expressed in grain units.

| parameter | Obtained value |
|---|---|
| $v_0$ [m/s] | 13.25±0.4 |
| $grain\ elasticity$ [grain units] | 0.18±0.02 |
| $grain\ friction$ [grain units] | 0.98±0.05 |

## S6. Final Considerations

The proposed methodology successfully integrates particle-based simulation and evolutionary optimization to model granular impact phenomena with high fidelity. Its flexibility and adaptability make it a promising tool for applications in geophysics, planetary science, and engineering involving granular materials.

**References**

He Lv, Qiguang He, Xiaowei Chen, Pengfei Han, "Numerical simulation of impact crater formation and distribution of high-pressure polymorphs", Acta Astronautica, Volume 203, 2023, Pages 169-186, ISSN 0094-5765,

Hetem, A. "The Search for Parameters and Solutions: Applying Genetic Algorithms on Astronomy and Engineering". In: Shangce Gao. (Org.). Bio-Inspired Computational Algorithms and Their Applications. 1ed.Rijeka, Croatia: InTech, 2012, v. 1, p. 161-186.

Howling Moon Software, "About Chipmunk2D", Howling Moon Software, December, 2024. [Online]. Available: http://chipmunk-physics.net/aboutChipmunk.php

Liang, H., He, S. & Liu, W. Dynamic simulation of rockslide-debris flow based on an elastic–plastic framework using the SPH method. Bull Eng Geol Environ 79, 451–465 (2020). https://doi.org/10.1007/s10064-019-01537-8

Press, W., Teukolsky, S., Vetterling, W., Flannery, B., 1995, “Numerical Recipes in C” 2nd Edition, (Cambridge, UK: Cambridge University Press)
