# Supplementary material for: Complex crater formation by low energy impactors
Source: PLoS One. 2025 Nov 6;20(11):e0326628. doi: 10.1371/journal.pone.0326628 (PMC12591455; doi:10.1371/journal.pone.0326628)
Supplement: Attachment — Submitted filename: ResponseLetter.pdf [file pone.0326628.s002.pdf]

Rodrigo Tardini Paulino
Center for the Physics of Materials
McGill University
Montreal, QC, Canada
July 25th, 2025

Response letter for a new submission to PLOS One

Dear Reviewers and Dr. Qin,

We thank the reviewers for the overall positive comments and constructive feedback. In this letter, we will address the concerns raised by the reviewers on the manuscript entitled "**Complex crater formation by low energy impactors**". The reviewers' remarks are shown in red and our responses are addressed individually in black.

Reviewer 1: The experiment presented is correctly and interestingly designed. The results obtained appear to be reliable. However, the paper lacks justification for the research undertaken. The authors have not explained the practical aspect of the proposed research. This is, in my opinion, a significant shortcoming of this manuscript.

Authors:

We thank the reviewer for their positive assessment of our experimental design and results. In response to the comment regarding the practical justification for our study, we have revised the Introduction section to clarify the broader relevance of our work, including potential applications.

Our study explores crater formation in low-energy impacts on layered granular beds, a regime that remains largely unexplored in the literature but is highly relevant in real-world scenarios. To highlight this, we added a discussion in the manuscript connecting our findings to a civil engineering practice commonly used for rockfall damage mitigation, and to the study of regolith thickness in planetary geology. Beyond these cases outlined in the manuscript, we appreciate this opportunity to elaborate further examples that are very commonly encountered, that didn't fit the scope of the manuscript:

Liquid impactors: Our experiments with water droplets on layered beds closely resemble common real-world phenomena, such as raindrop impacts on sandy or dusty surfaces that overlay hard infrastructure. For instance, in shoreline or arid environments, vehicles, buildings, and even sports equipment (like the beach tennis racket shown in the figure below) are often covered by a thin layer of sand or dirt. When rain or irrigation interacts with this surface, it creates crater-like structures similar to those we studied. This is of significance to many important natural, agricultural and industrial processes like soil erosion, drop irrigation, spray-coatings or forensics.

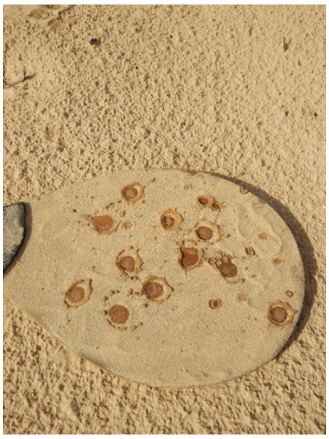

Fig. R1 – Craters formed from droplets onto a wood racquet covered in sand.

Solid impactors: Historical and contemporary examples from military applications also show how target inhomogeneity influences crater morphology. For instance, the Grand Slam bomb dropped in WWII on a fortified German facility resulted in a non-bowl-shaped crater due to the layered structure of the concrete. More recently, images of a Russian FAB-1500 bomb dropped in Ukraine show a flat-floored crater typical of a complex morphology, likely due to layered terrain properties. These real-world outcomes are consistent with the crater features reproduced in our laboratory experiments, thus the study of crater formation is of significance not only on planetary science, but also to applied impact engineering.

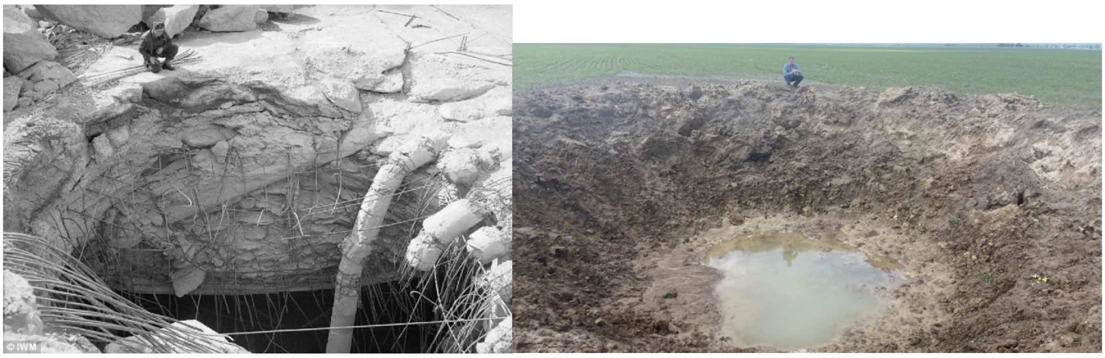

Figs. R2 – (left) Complex crater generated by the Grand Slam bomb in a German facility in 1945 (link). (right) Flat-floored crater generated by a Russian bomb in a rural region of Ukraine (link).

While we hope this clarifies the practical motivation and potential implications of our research, our primary motivation still is to advance the fundamental understanding of crater morphology and energy dissipation in layered systems. In the end, the ultimate practical purpose of a scientific project is to understand nature.

Reviewer 1: In addition, the literature review performed is not sufficient. The authors have not included an adequate reference to the work of previous researchers, either to outline the background to their own research or to support the results obtained.

Authors: We have added 15 relevant references to the manuscript, expanding the context for our study and reinforcing our interpretation of the results.

We also clarified how our work fits within the existing literature. After a thorough review, we found only one previous study using layered targets—Oberbeck and Quaide (1967)—which involved very high-energy solid projectiles, in a regime far removed from ours. We now include a direct comparison and highlight how our study explores a new, low-energy regime using layered targets, previously unaddressed.

Finally, we improved our discussion by drawing clearer comparisons with prior studies throughout the manuscript. These changes better establish the background and emphasize how our work extends current knowledge.

Reviewer 1: The most significant major shortcoming of this article is the discussion, which is conducted as a description of the results obtained rather than a polemic against the results of other researchers. I consider this to be a significant shortcoming that prevents the article from being published in its current form.

Authors:

We appreciate the reviewer's feedback and have rewritten the Results and Discussion sections to better emphasize comparisons with existing literature. In the revised manuscript, we now explicitly contrast our findings with previous experimental and theoretical work, including discussion of where existing models such as the Schmidt-Holsapple scaling succeed or break down when applied to our regime.

That said, we also wish to clarify that our experimental configuration—low-energy impacts on layered granular targets—represents a new and largely unexplored regime. As a result, direct parallels with published results are limited, and many models developed for homogeneous targets or high-energy impacts are not expected to apply. We now make this point more clearly in the manuscript.

Despite this, we have strengthened the discussion by clearly establishing where our results deviate from known scaling laws and by contextualizing those deviations as evidence of the novel energy dissipation dynamics introduced by layering. These changes aim to better highlight both the novelty and relevance of our findings while addressing the reviewer's concern.

Reviewer 2: In this regard what you have to do while resubmitting is that you have show real examples for your experiment. If you are not getting examples from Earth, you can choose Moon or Mars.

Authors: We thank the reviewer for their positive assessment of our experimental design and results. In response to the comment regarding the practical justification for our study, we have revised the Introduction section to clarify the broader relevance of our work, including potential applications.

Our study explores crater formation in low-energy impacts on layered granular beds, a regime that remains largely unexplored in the literature but is highly relevant in real-world scenarios. To highlight this, we added a discussion in the manuscript connecting our findings to a civil engineering practice commonly used for rockfall damage mitigation, and to the study of regolith thickness in planetary geology. Beyond these cases outlined in the manuscript, we appreciate this opportunity to elaborate further examples that are very commonly encountered, that didn't fit the scope of the manuscript:

Liquid impactors: Our experiments with water droplets on layered beds closely resemble common real-world phenomena, such as raindrop impacts on sandy or dusty surfaces that overlay hard infrastructure. For instance, in shoreline or arid environments, vehicles, buildings, and even sports equipment (like the beach tennis racket shown in the figure below) are often covered by a thin layer of sand or dirt. When rain or irrigation interacts with this surface, it creates crater-like structures similar to those we studied. This is of significance to many important natural, agricultural and industrial processes like soil erosion, drop irrigation, spray-coatings or forensics.

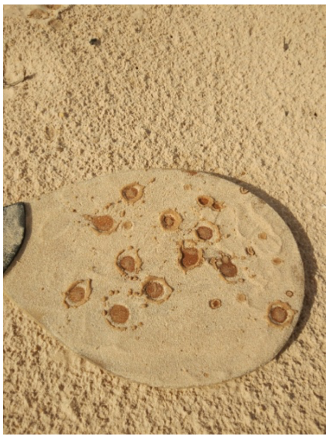

Fig. R1 – Craters formed from droplets onto a wood racquet covered in sand.

Solid impactors: Historical and contemporary examples from military applications also show how target inhomogeneity influences crater morphology. For instance, the Grand Slam bomb dropped in WWII on a fortified German facility resulted in a non-bowl-shaped crater due to the layered structure of the concrete. More recently, images of a Russian FAB-1500 bomb dropped in Ukraine show a flat-floored crater typical of a complex morphology, likely due to layered terrain properties. These real-world outcomes are consistent with the crater features reproduced in our laboratory experiments, thus the study of crater formation is of significance not only on planetary science, but also to applied impact engineering.

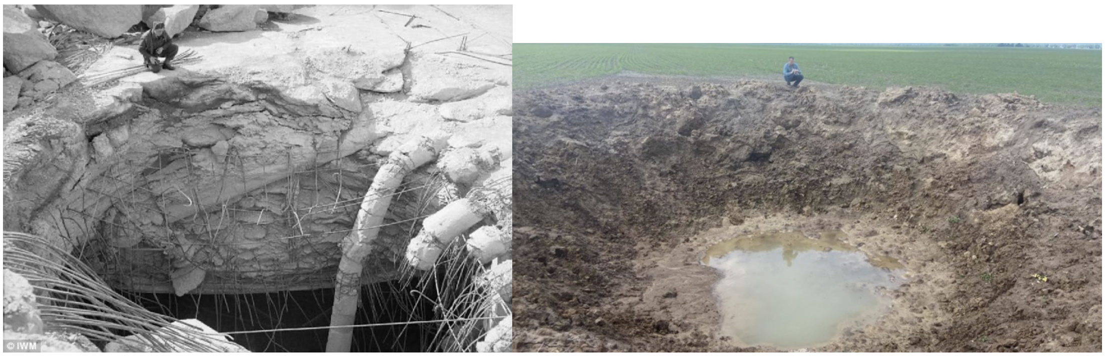

Figs. R2 – (left) Complex crater generated by the Grand Slam bomb in a German facility in 1945 (link). (right) Flat-floored crater generated by a Russian bomb in a rural region of Ukraine (link).

Furthermore, we would like to highlight the planetary examples already outlined throughout the text that we couldn't add images to the manuscript:

- The Barringer Crater on Earth shows a square-shaped outline due to pre-existing fault structures across the impact site.

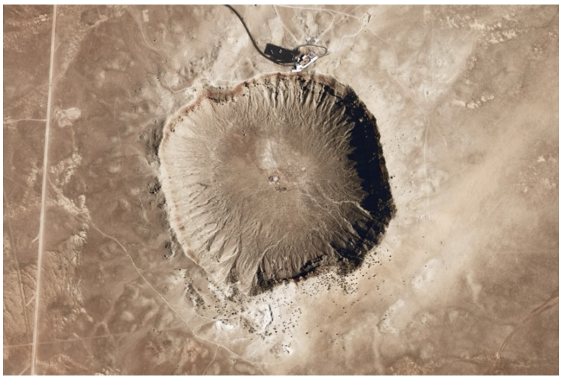

Figs. R3 – Barringer crater, from NASA Earth Observatory.

- Large multi-ring basins such as Chicxulub and Orientale have been modeled using layered target assumptions to account for their complex morphology.

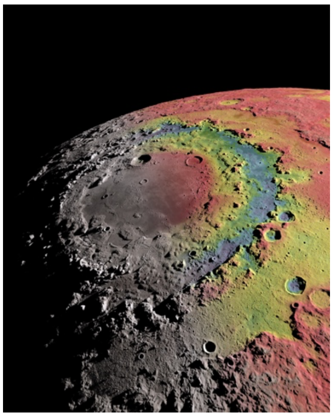

Figs. R4 – Orientale basin on the moon, from NASA.

- On Mars, craters with flat floors and central peaks—morphologies we reproduce in the lab—have been linked to impacts in layered or icy subsurfaces.

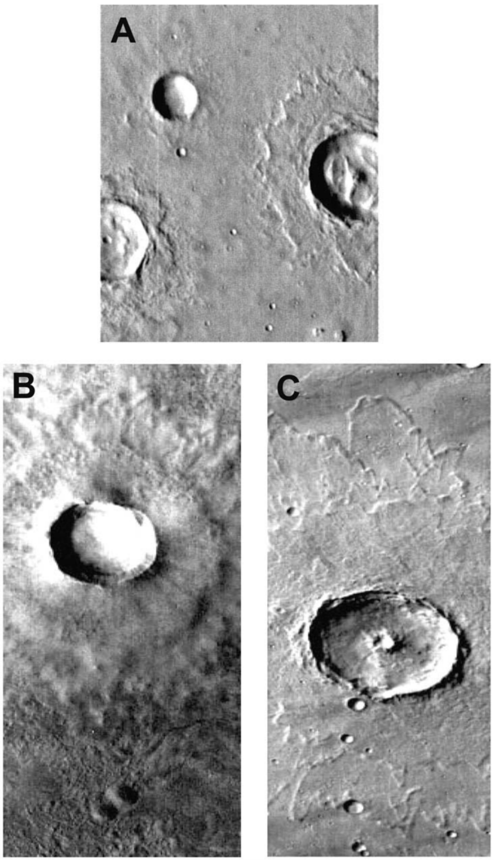

Fig. R5. Example images of craters in Mars. (A) Crater on the right is 12 km in diameter and located near 24°N 101°E (THEMIS image I02493005). (B) Crater is 17 km in diameter and located near 49°N 230.5°E (THEMIS image I03350005). (C) Crater is 25 km in diameter and located 6°N 304°E (THEMIS image I03218002). Taken from (Barlow *et al.,* 2005).

- On the Moon, several studies have used crater shapes to infer the presence and distribution of regolith and subsurface structures.

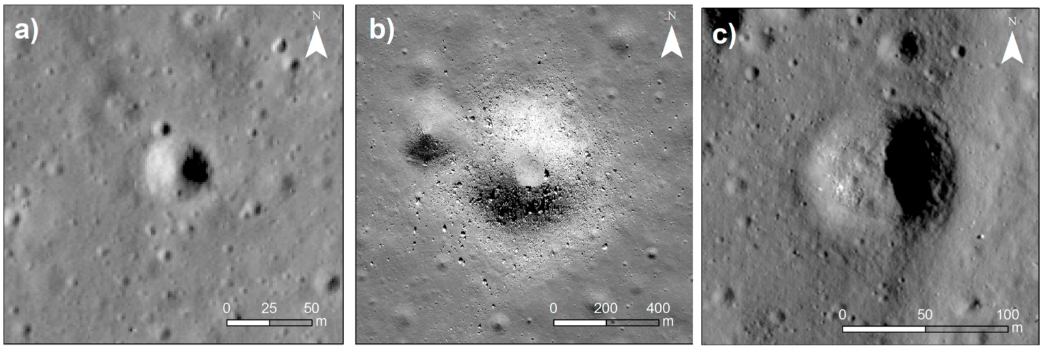

Fig. R6. Example images of craters in the lunar maria, taken with the Narrow Angle Camera aboard the Lunar Reconnaissance Orbiter. The different morphologies are (A) simple crater (16.94°N, 60.01°E), (b) central-peak (58.96°N, 8.12°W), (c) flat-bottomed (19.69°S, 127.17°E), Taken from (Prieur *et al.,* 2018).

We hope this clarifies the practical motivation and implications of our work.

On behalf of all authors,

Rodrigo T. Paulino.
