# Supplementary material for: Complex crater formation by low energy impactors
Source: PLoS One. 2025 Nov 6;20(11):e0326628. doi: 10.1371/journal.pone.0326628 (PMC12591455; doi:10.1371/journal.pone.0326628)
Supplement: Attachment — Submitted filename: ResponseLetter_auresp_2.pdf [file pone.0326628.s003.pdf]

Rodrigo Tardini Paulino
Center for the Physics of Materials
McGill University
Montreal, QC, Canada
September 22nd, 2025

Response letter for a new submission to PLOS One

Dear Reviewer and Dr. Qin,

We thank the reviewer for the positive comments and constructive feedback. In this letter, we will address the comments made by the reviewer on the manuscript entitled "**Complex crater formation by low energy impactors**". The reviewers' remarks are shown in red and our responses are addressed individually in black.

Reviewer 1: Due to the diversity of the experiments conducted and numerical simulations, the text is still difficult to fully understand in places. I therefore suggest introducing a division based on the impactor used: i) liquid, ii) solid, iii) granular, both in the description of the methodology and in the Results and Discussion. In my opinion, this minor change in the text and figures will significantly improve the readability of the article.

Authors: We have added subsections separating the liquid, solid and granular impactors in the methodology and discussion sections. Due to the similarities in the methodology and crater diameter dependencies, we have opted to discuss the solid and granular impacts together in these sections.

Reviewer 1: Please remove from the text your own descriptions (well understood by the author, but difficult for the reader to understand unambiguously), such as: described as an inverted rim placed inside the outer rim (256).

Authors: This is a situation where attempting to provide further description only made understanding the phenomena more difficult, so we thank the reviewer for this input. We have removed such descriptions from the main text.

Reviewer 1: Please also add a statistical analysis of the results obtained.

Authors: We have included reduced chi-squared analysis for all models employed in the manuscript, as can be seen in the captions of Figures 2a, 2b and 3a.

It is important to point out, however, that the statistical relevance of our results was always accounted for by performing the experiments several times, and considering the standard deviation of the mean as the experimental error, as described in the Experimental Methodology and shown explicitly in all data points. These uncertainties were always

considered in the fitting procedures. Accordingly, all reported parameters are shown with their respective uncertainties, using the parentheses convention, i.e., 0.170(9) is the same as 0.170 ± 0.009.

On behalf of all authors,

Rodrigo T. Paulino.
